# Disruption of the AKT/MTOR pathway by *Leishmania major* promastigotes

**Published:** 2011-01-10

**Authors:** Christine Matte, Albert Descoteaux

**Affiliations:** 1 INRS-Institut Armand-Frappier, Laval, Quebec, Canada, H7V 1B7

## 

Signaling through the Akt/mammalian target of rapamycin (mTOR) pathway plays a pivotal role in the regulation of multiple cellular processes, including proliferation, apoptosis, protein synthesis and autophagy. It is therefore a major target of microbial infections and tumors. Protozoa of the *Leishmania* genus cause a wide spectrum of diseases in humans, termed leishmaniases, with clinical manifestations ranging from self-healing skin ulcers to life-threatening visceral disease. These parasites primarily infect macrophages and are renowned for their ability to sabotage host-cell signal transduction pathways. Here, we report that infection of Balb/c bone marrow-derived macrophages with the promastigote stage of *Leishmania major* results in rapid, time-dependent degradation of key components of the Akt/mTOR axis, including Akt, mTOR and the tuberous sclerosis complex-2 (TSC-2). Disruption of the Akt/mTOR pathway by *L. major* is dependent on the surface metalloprotease gp63, an important virulence factor of the parasite, and appears to be strain- and species-specific. The consequences of the degradation of key intermediates in the Akt/mTOR pathway on downstream responses are currently being investigated. These studies highlight a novel mechanism by which *L. major* interferes with macrophage functions and responses and will provide a better understanding of *Leishmania* pathogenesis.

